# Supplementary material for: Towards unified AI-driven fracture mechanics: the extended deep energy method (XDEM)
Source: Nat Commun. 2026 Aug 15;17:8492. doi: 10.1038/s41467-026-76748-1 (PMC13484471; doi:10.1038/s41467-026-76748-1)
Supplement: Supplementary file 3 [file 41467_2026_76748_MOESM3_ESM.zip › pic/Results/Stress_intensity_factor/Cross/FEM/beta_30/u1_displacement.pdf]

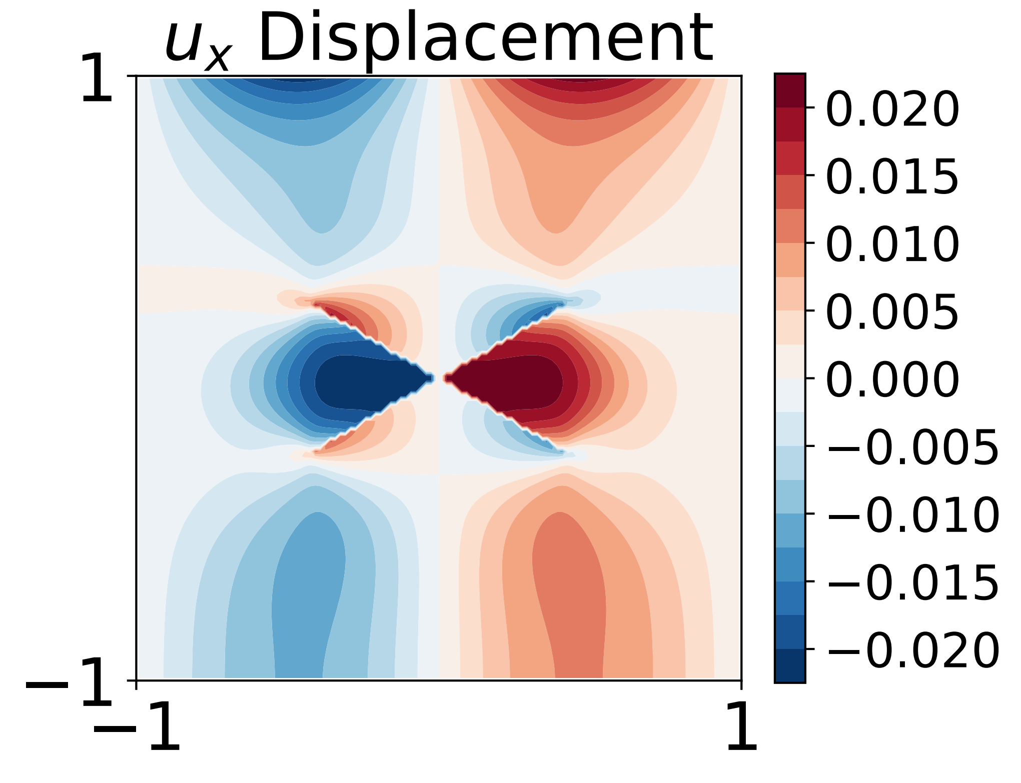

u_x Displacement
1
−1
−1
1
0.020
0.015
0.010
0.005
0.000
−0.005
−0.010
−0.015
−0.020
